# Overnutrition in Infants Is Associated With High Level of Leptin, Viral Coinfection and Increased Severity of Respiratory Infections: A Cross-Sectional Study

**DOI:** 10.3389/fped.2020.00044

**Published:** 2020-02-18

**Authors:** Guisselle Arias-Bravo, Gustavo Valderrama, Jaime Inostroza, Marjorie Reyes-Farías, Diego F. Garcia-Diaz, Francisco Zorondo-Rodríguez, Loreto F. Fuenzalida

**Affiliations:** ^1^Facultad de Ciencias de la Salud, Instituto de Ciencias Biomédicas, Universidad Autónoma de Chile, Santiago, Chile; ^2^Instituto de Ciencias de la Salud, Universidad de O'Higgins, Rancagua, Chile; ^3^Urgencia Materno-Infantil, Clínica Dávila, Santiago, Chile; ^4^Jeffrey Modell Centre for Diagnosis and Research in Primary Immunodeficiencies, Facultad de Medicina, Universidad de La Frontera, Temuco, Chile; ^5^Laboratorio de Nutrigenomica, Departamento de Nutricion, Facultad de Medicina, Universidad de Chile, Santiago, Chile; ^6^Departamento de Gestión Agraria, Facultad Tecnológica, Universidad de Santiago de Chile, Santiago, Chile

**Keywords:** overnutrition, children, viral respiratory infection, severity, viral coinfection, leptin

## Abstract

**Objective:** To investigate the relationship of overnutrition (obese and overweight) with severity of illness in children hospitalized with acute lower respiratory infections (ALRIs), frequency of viral coinfections and leptin levels.

**Methods:** We studied 124 children <2 years old that were hospitalized for ALRI. Nutritional status was calculated by z-scores according to weight-for-age z-scores, length or height-for-age z-scores, and weight-for-height z-scores. Nasopharyngeal aspirates (NPAs) were obtained and viral respiratory pathogens were identified using reverse transcription polymerase chain reactions (RT-PCR). Respiratory syncytial virus (RSV) load was assessed using quantitative RT-PCR. NPA and plasma leptin level were measured. Clinical data and nutritional status were recorded, and patients were followed up until hospital discharge. Viral coinfection was defined as the presence of two or more viruses detected in the same respiratory sample. Severity of illness was determined by length of hospitalization and duration of oxygen therapy.

**Results:** Children with overnutrition showed a greater frequency of viral coinfection than those with normal weight (71% obese vs. 37% normal weight *p* = 0.013; 68% overweight vs. 37% normal weight *p* = 0.004). A lower RSV load was found in obese (5.91 log_10_ copies/mL) and overweight children (6.49 log_10_ copies/mL) compared to normal weight children (8.06 log_10_ copies/mL; *p* = 0.021 in both cases). In multivariate analysis, obese, and overweight infants <6 months old were associated with longer hospital stays (RR = 1.68; CI: 1.30–2.15 and obese: RR = 1.68; CI: 1.01–2.71, respectively) as well as a greater duration of oxygen therapy (RR = 1.80; IC: 1.41–2.29 and obese: RR = 1.91; CI: 1.15–3.15, respectively). Obese children <6 months showed higher plasma leptin level than normal weight children (7.58 vs. 5.12 ng/μl; *p* <0.046).

**Conclusions:** In infants younger than 6 months, overnutrition condition was related to increased severity of infections and high plasma leptin level. Also, children with overnutrition showed a greater frequency of viral coinfection and low RSV viral load compared to normal weights children. These findings further contribute to the already existent evidence supporting the importance of overnutrition prevention in pediatric populations.

## Introduction

Acute lower respiratory tract infections (ALRIs) are a common illness in children <5 years old, with significant morbidity and mortality in infants and young children under the age of two (1–3). Respiratory syncytial virus (RSV) is the most important viral pathogen causing ALRIs in young children (4).

There are many risk factors related to ALRIs. Some of these are relative to pathogens, such as viral load (5), while others are directly associated with the host, such as prematurity, inadequate breastfeeding, and undernutrition (6). The influenza AH1N1 2009 pandemic showed, for the first-time, that obesity is a risk factor for the severity of ALRIs in adults (7, 8). Pediatric obesity has also been described as a risk factor for lower respiratory tract infections, as well as the severity and morbidity of these infections (9). However, the empirical evidence needed to estimate the impact of overnutrition (including overweight and obese conditions) on the severity of viral respiratory infections in children is still lacking (10).

Overnutrition causes an excessive accumulation of body fat. It is a highly prevalent condition and has been ranked as the foremost epidemic of the twenty-first century (11). Obesity results in a chronic state of inflammation with systemic implications for immunity (12). Leptin, cytokine-like hormone that is positively correlated with the body mass index, mediates upregulation of suppressor of cytokine signaling (SOCS) proteins (13). SOCS proteins are involved in the negative regulation of Janus-activated kinase–signal transducer and activator of transcription (JAK-STAT) signaling and the induction of type I and type II IFNs and pro-inflammatory cytokines, suggesting a potential mechanism by which respiratory viruses response in obesity may be attenuated (8, 14, 15).

In addition to immune modulation of obesity, alterations in membrane lipid composition have been observed in erythrocyte from obese children. The membrane composition has also revealed an increase in cholesterol content as has the cholesterol to phospholipid molar ratio, both of which have been positively correlated with a decrease in membrane fluidity, body-mass index, and plasma cholesterol levels (16). Cholesterol is a critical structural component of lipid rafts, key structures in binding and endocytosis of respiratory viruses (17). In accordance with this, it could be expected that the membrane of obese children's respiratory cells may undergo some type of modification that could benefit the entry of respiratory viruses.

Hence, the objective of this study was to estimate the relationship of overnutrition on severity of illness in infants (aged between 0 and 5 months) and children (aged between 6 and 24 months) hospitalized with ALRIs. Moreover, frequency of viral coinfection, RSV viral load and levels of leptin according to nutritional status were evaluated.

## Materials and Methods

### Study Population and Nutritional Assessment

A cross-sectional study was conducted for 2 consecutive epidemic periods (from May to August) in 2016 and 2017. Patients were selected based on following inclusion criteria: children <24 months old that were hospitalized for ALRI at two medical centers in Santiago, Chile: Urgencia Materno-Infantil at Clínica Dávila and Dr. Exequiel González Cortés Hospital. Patients were classified as having bronchiolitis, bronchitis or pneumonia. Diagnoses were made in patients with dyspnea, signs of lower respiratory tract infections (wheezing, retractions) and/or a positive chest x-ray (infiltrates, atelectasis and air trapping). The clinical and demographic characteristics of each patient were also recorded. The exclusion criteria were: children with nutritional risk and/or undernourished children, newborns <28 days old, patients with a diagnosis of recurrent wheezing, previous hospitalization for any cause, primary or secondary immunodeficiency, gestational age of <37 weeks, bronchopulmonary dysplasia, previous mechanical ventilation, congenital heart disease, respiratory infection during the previous 2 weeks, corticosteroid intake 72 h before sample was taken, and children with negative samples for respiratory viruses. Figure S1 shows exclusion criteria. Children were classified into three groups according to their nutritional status (NS) using WHO Anthro 2011 v.3.2.2 program: normal weight, overweight, and obese. NS was determined by z-scores according to the following anthropometric indicators: weight-for-age z-scores, length or height-for-age z-scores, and weight-for-height z-scores. Normal weight was defined as −0.9 to 0.9 SD, overweight as 1.0 to ≤2.0 SD and obese as >2 SD. Undernourished children and those with nutritional risk were defined with a weight-for-age z-score and weight-for-height z-score of <1 SD, below the mean, and were therefore excluded from the study. The weight of each patient was measured at hospital admission. Disease severity was assessed by using the standard criteria of length of hospitalization and duration of treatment with supplemental oxygen (18, 19).

### Nasopharyngeal Aspirate Sample Collections

Approximately 3 ml of nasopharyngeal aspirate (NPA) were collected from each patient, usually during the admission process (<3 h) and always within the first 24 h of hospitalization. Secretions from both nostrils were aspirated without flushing using a soft catheter placed in a collection trap with 3 ml of sterile saline solution and immediately transported on ice to the laboratory. Aliquots of 2 ml were stored at −80°C for viral analysis. Aliquots of 1 mL were centrifuged at 1,000 g for 15 min at room temperature and the supernatants obtained were stored at −80°C until determination of leptin level.

### Viral Analysis

Viral RNA and DNA were simultaneously extracted from 150 μL of the NPA samples using the Viral RNA isolation kit NucleoSpin (Macherey-Nagel®, Düren, Germany) following the manufacturer's instructions, and stored at −80°C, until use. Viral infection was detected with real-time PCR kit ARGENE® (bioMérieux, Marcy-I′Étoile, France), for RSV, human metapneumovirus (HMPV), parainfluenza virus 1–4 (HPIV), human coronavirus (HCoV), adenovirus (AdV), bocavirus (HBoV), influenza A (FluA), influenza B (FluB), and rhinovirus/enterovirus (HRV/HEV), following the manufacturer's instructions. Viral co-infection was defined as the presence of two or more viruses detected in the same respiratory sample.

### RSV Viral Load Quantification

Forty-five patients that tested positive for RSV were selected using the ARGENE® real-time PCR kit. A new aliquot was used with 150 μL of NPA. Nucleic acids were extracted using the Viral RNA isolation kit NucleoSpin (Macherey-Nagel®, Düren, Germany). The qRT-PCR was performed using the Takyon™ Rox Probe MasterMix dTTP blue kit (Eurogentec, USA), using primers with N1 (5′-GGAACAAGTTGTTGAGGTTTATGAATATGC3′) and N2 probes (CTTCTGCTGTCAAGTCTAGTACACTGTAGT-3′) (20). The conditions of the PCR reaction were: preincubation at 95°C for 3 min; 40 cycles at 95°C for 10 s and 60°C for 30 s. Threshold cycles of positive samples for RSV were compared with the standard of curves generated by the amplification of known plasmid copy numbers of the pGEM-T DNA (Promega) containing primer targets. In order to determine the effective amount of copies of viral DNA molecules contained in each sample of the endogenous RNAse P gene, the TaqMan® RNase P kit (Applied Biosystems, Woolston, UK) was used. The Cq values were interpolated and normalized according to the following formula: Cq normalized sample = (Cq viral sample) × (Cq RNase P of the sample)/average value of Cq of all the RNase P samples) (21). RSV quantification was reported as log_10_ copies/mL.

### Blood Samples

About 2 ml of blood were collected in sodium heparin collection tubes (BD Vacutainer) usually during the admission process. The sample was centrifuged at 1,000 g for 15 min at room temperature. The plasma obtained was divided into aliquots and stored at −80°C.

### Quantification of Plasma and NPA Leptin Level

Leptin concentration in plasma and NPA was measured using the Magnetic Luminex®assay (R&D), according to manufacturer protocol.

### Statistical Analysis

Descriptive analyses, medians (ranges), and frequency distributions were used to summarize the demographic and baseline attributes of patients. Associations among the dependent and independent variables were assessed by either the χ^2^, Fisher's exact or Mann–Whitney tests. Spearman's test was used to estimate the association between viral load and severity. Multivariate Poisson regression models were used to determine the factors that predict severity of infection (length of stay and duration of oxygen therapy). Multicollinearity analyses among the independent variables were performed and none were observed. The cutoff point for multicollinearity was determined as variance inflation factor <5 and tolerance >0.2. We did not include birth weight in the multivariate regression model because it increases the multicollinearity in the model (Variance Inflation Factors from 2.32 to 6.47). The relative risk (RR) for increased length of stay and oxygen therapy was calculated by taking the estimated Poisson regression coefficient (β) for each variable and transforming it by eβ [exp^*^confidence interval] of each independent variable for the model. Statistical significance was set at *p* < 0.05 for all analyses, along with a 95% confidence interval (CI). All analyses were performed with Stata 14.1 software (Statacorp, Texas, USA).

## Results

### Overnutrition Is Associated With High Frequency of Viral Coinfection and Low Viral Load of RSV

A total of 160 children were screened, and 124 patients were eligible for this study (Figure S1). The clinical and demographic characteristics of patients are presented in Table 1. Obesity was found in 13.7% of the patients, 25.8% were overweight, and 60.5% presented normal weights. Most of the children had both breastfeeding and complete vaccination schedules. The principal diagnosis was pneumonia (55.6%), followed by bronchiolitis and bronchitis (Table 1).

**Table 1:** Summary of clinical and demographic data of children hospitalized with acute respiratory infections.

**Demographic and clinical features**	
Total	*N* = 124
Origin *N* (%)	
Dr. Exequiel González Cortés Hospital	41 (33.1)
Maternal and Child Urgency, Clínica Dávila	83 (66.9)
Gender *N* (%)	
Male	64 (51.6)
Female	60 (48.4)
Age (month), median (range)	7 (1–21)
Nutritional status *N* (%)	
Normal weight	75 (60.5)
Overweight	32 (25.8)
Obese	17 (13.7)
Birth weight^a^	3.4 (2.0–4.9)
Breastfeeding^b^ *N* (%)	
Yes	82 (67.2)
No	40 (32.8)
Vaccines^c^ *N* (%)	
Yes	104 (84.6)
No	19 (15.4)
Diagnosis^c^ *N* (%)	
Pneumonia	69 (55.6)
Bronchiolitis	37 (29.8)
Bronchitis	18 (14.5)
Management, median (range)	
Days of hospitalization	6 (1–29)
Days of oxygen therapy^c^	5 (0–26)
Assistan ventilation	16 (12.9)

*Data are presented as median values (minimum–maximum) or no. (%) of subjects (percentages are calculated for children with complete data*.

a *N = 116*,

b *N = 122*,

c *N = 123)*.

**Figure 1:**
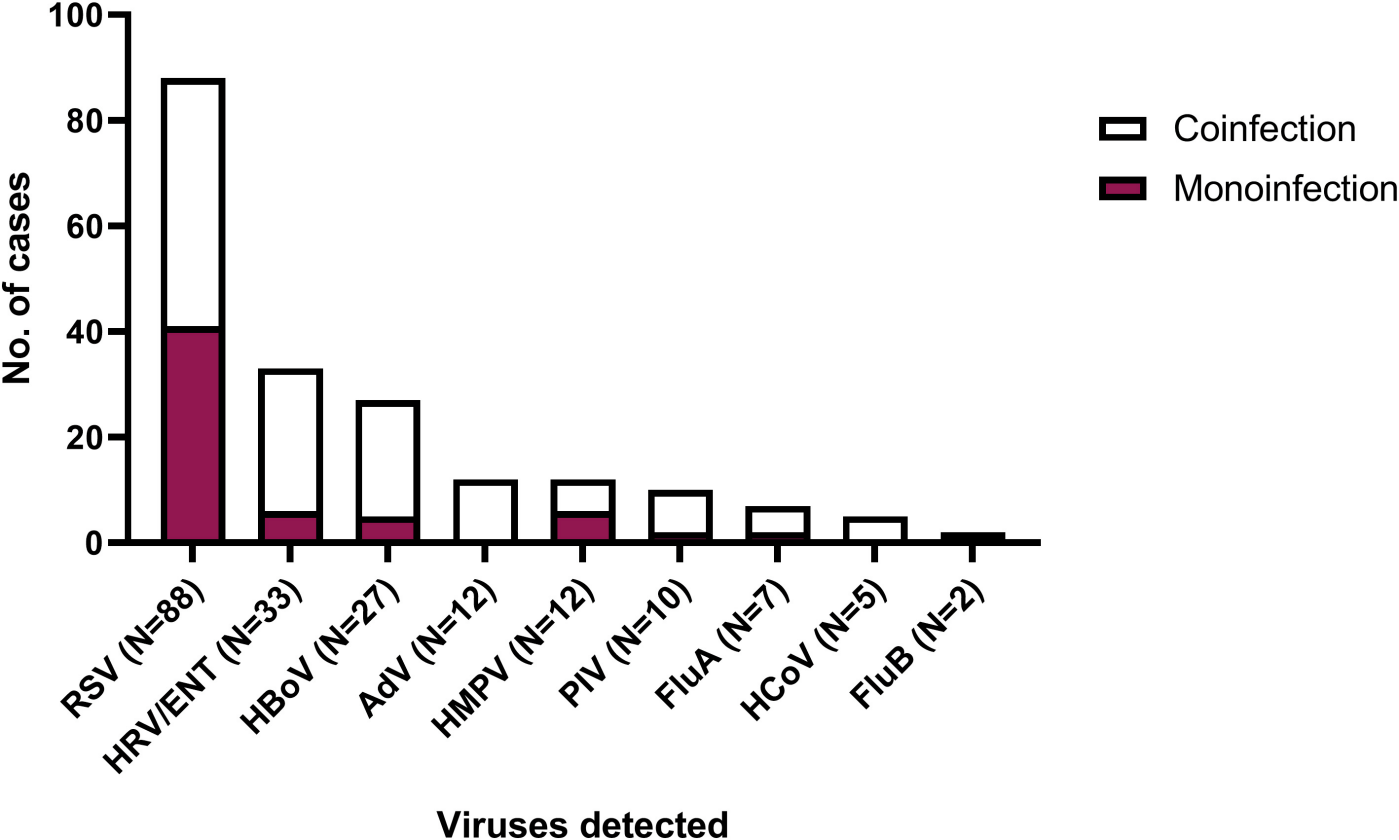
Number of cases with monoinfection and viral coinfection. RSV, respiratory syncytial virus; HBoV, bocavirus; HCoV, coronavirus; HRV/ENT, rhinovirus/enterovirus; PIV, parainfluenza; AdV, adenovirus; HMPV, human metapneumovirus; FluA, influenza A; FluB, influenza B.

A total of 63 (50.8%) patients tested positive for a single respiratory virus, while another 61 patients (49.2%) tested positive for more than one respiratory virus. Of these, 50 (82.0%) tested positive for two viruses and 11 (18.0%) for three viruses. RSV was the most frequently detected virus in 88 cases (70.9%), followed by HRV/HEV in 33 (26.6%) and HBoV in 27 (21.7%). Most viruses were found in coinfection (Figure 1). No differences were found between NS and type of virus detected (Figure S2).

**Figure 2:**
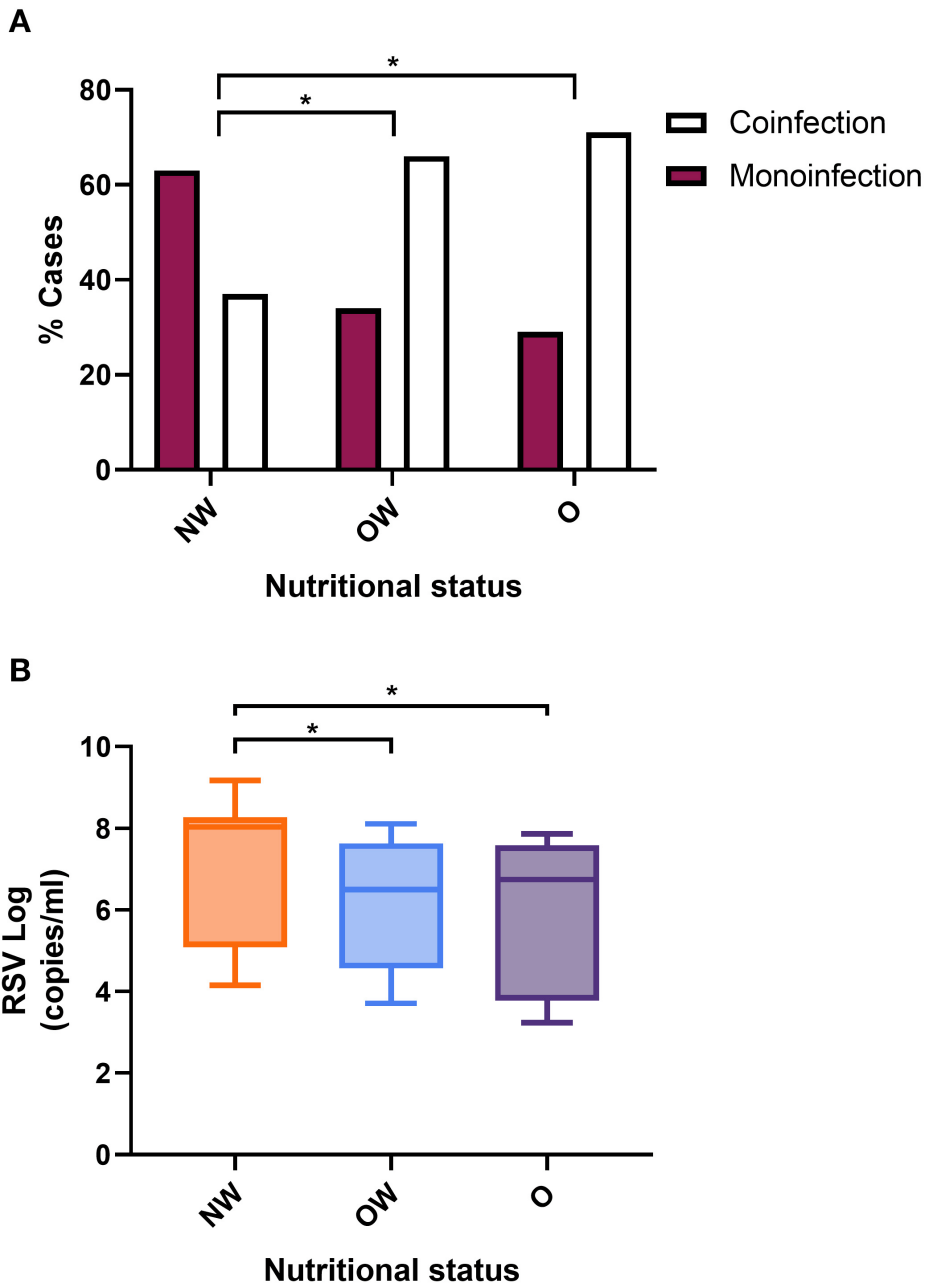
Frequency of viral coinfection and RSV viral load according to nutritional status. **(A)** Monoinfections and viral coinfections according to nutritional status. The values were analyzed by the Chi-squared test. *p* < 0.05 was significant. **(B)** RSV viral load vs. nutritional status. The data were normalized with the reference gene RNase P, with the following formula: Normalized sample Ct = (Ct sample) × (Ct RNase P of the sample)/average Ct value of all RNase P). The values were analyzed by the Mann–Whitney *U*-test. *p* < 0.05 was significant. NW, normal weight; OW, overweight; O, obese. *Significant levels at <5%.

Nutritional status and coinfection (≥2 viruses) were evaluated to assess the effect of overnutrition in viral coinfection. Obese and overweight infants showed a greater frequency of coinfection (obese 71% and overweight 68%) than those with normal weights (37%) (*p* = 0.013 and *p* = 0.004 respectively) (Figure 2A). No significant differences were found between overweight and obese infants (*p* = 0.839).

The effect of obesity and overweight conditions on the RSV load was assessed in 46 infants (10 obese, 14 overweight, and 22 normal weight) with available nasopharyngeal samples. The median of RSV loads in obese infants was 5.91 log_10_ copies/mL (min: 3.22 log_10_ copies/mL and max: 7.86 log_10_ copies/mL) and 6.49 log_10_ copies/mL (min: 3.71 log_10_ copies/mL and max: 8.11 log_10_ copies/mL) for overweight infants, while those with normal weights had a median of 8.06 log_10_ copies/mL (min: 4.15 log_10_ copies/mL and max: 9.17 log_10_ copies/mL). The difference between obese and overweight infants compared to those with normal weights was significant (obese *p* = 0.0205 and overweight *p* = 0.0212, respectively) (Figure 2B). No significant differences were found between overweight and obese infants (*p* = 0.6395). No correlation was evidenced between RSV load and length of hospital stay (r = −0.124; *p* = 0.411) or days of oxygen therapy (r = −0.097; *p* = 0.517).

### Obese and Overweight Children Less than 6 Months Show a Greater Severity of Respiratory Infection

Tables 2, 3 present the results of the multiple Poisson regression by transforming beta coefficients from the regression into relative risk calculations, which allowed us to test and evaluate the hypothesis that overnutrition is associated with the severity of viral respiratory infections, by adjusting covariates. When studying all of the patients, we found that only an overweight condition proved to be associated with a greater severity of infection both in length of stay (Table 2) (RR_OW:NW_ = 1.27; CI: 1.18–1.34) and duration of oxygen therapy compared with normal weight children (Table 3) (RR_OW:NW_ = 1.34; CI: 1.29–1.40). However, when we studied patients under and over 6 months separately, we found that both obesity and overweight conditions were associated with a greater length of stay (Table 2) (RR_OW:NW_ = 1.68; CI: 1.30–2.15 and RR_O:NW_ = 1.68; CI: 1.01–2.71) and duration of oxygen therapy compared with normal weight (Table 3) (RR_OW:NW_ = 1.80; CI: 1.41–2.29 and RR_O:NW_ = 1.91; CI: 1.15–3.15). These results show that obesity and overweight conditions are significantly associated with the severity of viral respiratory infections in very young children.

**Table 2:** Poisson multiple regression assessing the association of length of stay and nutritional status.

	**Length of stay**		
**Explanatory variables**	**All infants (*n* = 121) RR (95% CI)**	**Infants <6 months (*n* = 52) RR (95% CI)**	**Infants ≥6 months (*n* = 69) RR (95% CI)**
Overweight (OW:NW)	1.27 (1.18–1.34)^**^	1.68 (1.30–2.15)^**^	1.03 (0.92–1.13)
Obese (O:NW)	1.05 (0.86–1.43)	1.68 (1.01–2.71)^*^	0.82 (0.64–1.05)

Risk for increased hospitalization was calculated by taking the estimated Poisson regression coefficient (β) for each variable and transforming it by eβ [exp*confidence interval] of each independent variable for the model adjusted for age, gender, and breastfeeding. RR, relative risk; O, Obese; OW, overweight; NW, normal weight. In parenthesis, confidence interval at 95%.

*, ** *refer to significant levels at <5% and <1%. N = 121*.

**Table 3:** Poisson multiple regression assessing the association of duration of oxygen therapy and nutritional status.

	**Duration of oxygen therapy**		
**Explanatory variables**	**All infants (*n* = 121) RR (95% CI)**	**Infants <6 months (*n* = 52) RR (95% CI)**	**Infants ≥6 months (*n* = 69) RR (95% CI)**
Overweight (OW:NW)	1.34 (1.29–1.40)^**^	1.80 (1.41–2.29)^**^	1.06 (0.97–1.16)
Obese (O:NW)	1.18 (0.92–1.53)	1.91 (1.15–3.15)^*^	0.83 (0.61–1.13)

Risk for increased hospitalization was calculated by taking the estimated Poisson regression coefficient (β) for each variable and transforming it by eβ [exp*confidence interval] of each independent variable for the model adjusted for age, gender, and breastfeeding. RR, relative risk; O, Obese; OW, overweight; NW, normal weight. In parenthesis, confidence interval at 95%.

*, ** *refer to significant levels at <5% and <1%. N = 121*.

Also, we analyzed whether assisted ventilation is related to nutritional status and our results suggest that assisted ventilation, as a proxy of severity, was not statistically associated with overnutrition (Fisher's exact, *p* = 0.862).

### In Children Under 6 Months of Age, Children With Obesity Have Higher Plasma Leptin Levels than Children With Normal Weight

**Figure 3:**
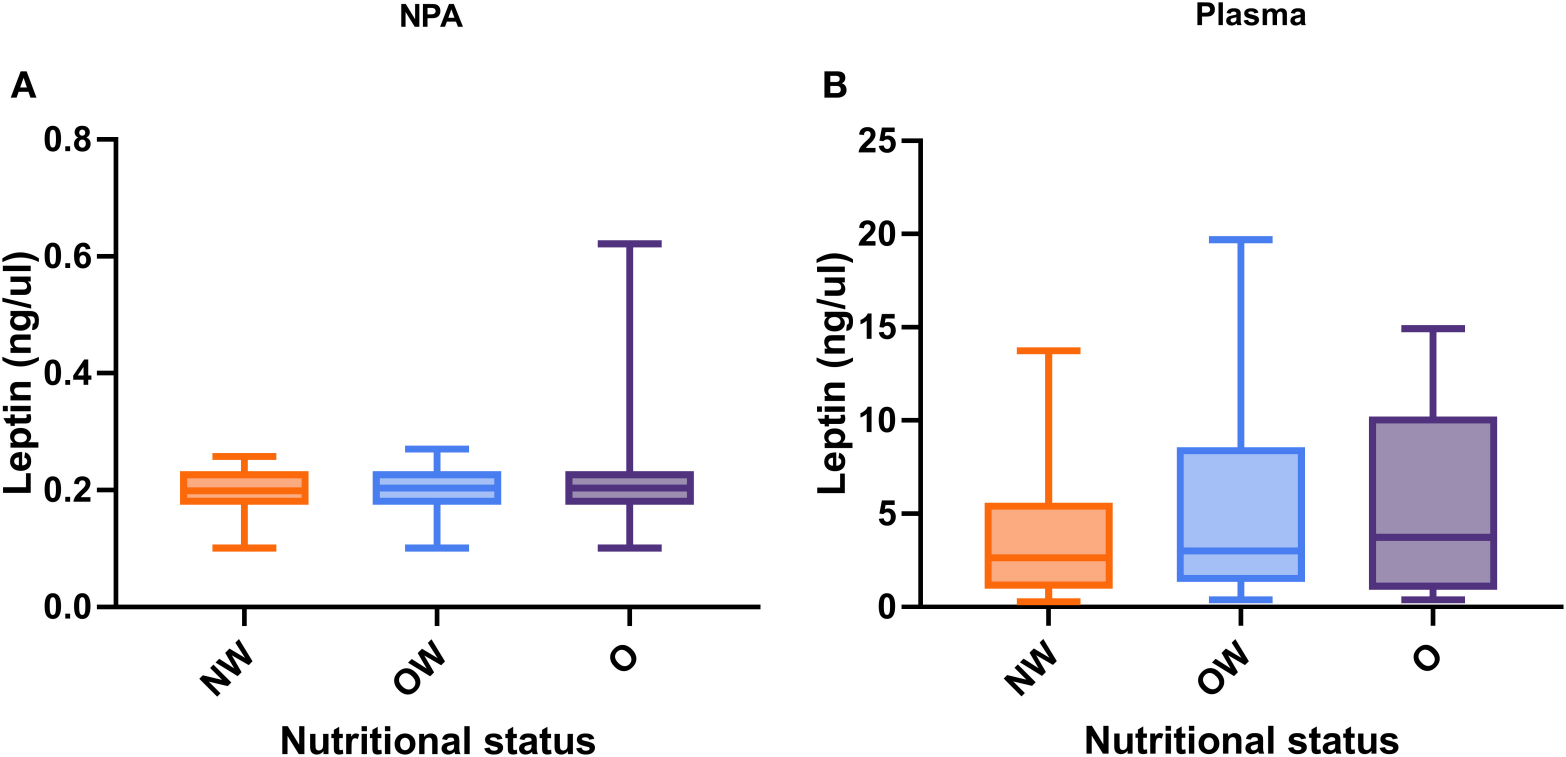
Leptin level according to nutritional status in NPA and plasma samples. **(A)** NPA samples **(B)** Plasma samples. The values were analyzed by the Mann–Whitney *U*-test. *p* < 0.05 was significant. NPA, nasopharyngeal aspirate; NW, normal weight; OW, overweight; O, obese.

**Figure 4:**
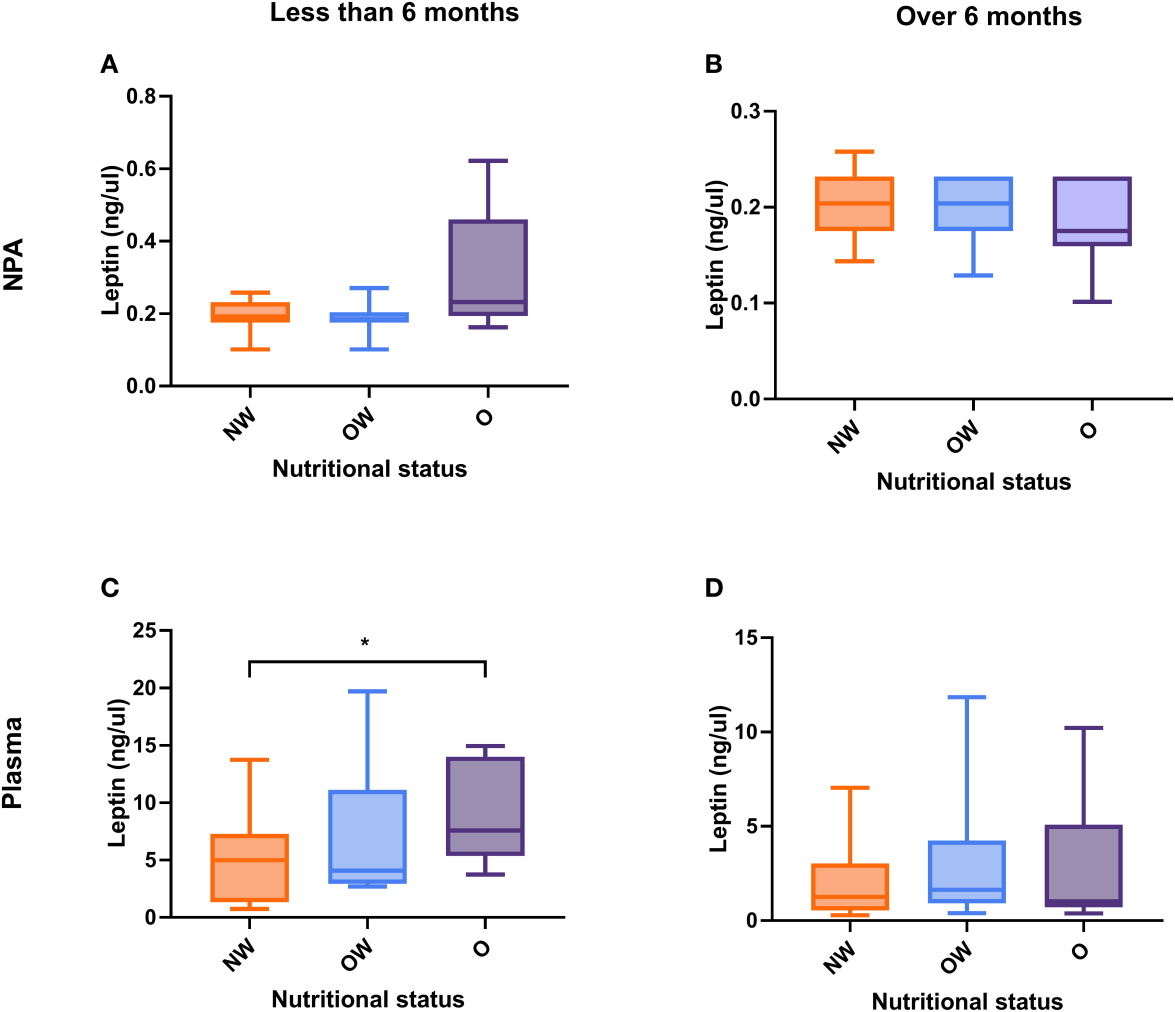
Leptin level in NPA and plasma samples according to nutritional status and age. **(A)** NPA level in children <6 months. **(B)** NPA level in children over 6 months. **(C)** Plasma level in children <6 months. **(D)** Plasma level in children over 6 months. The values were analyzed by the Mann–Whitney *U*-test. *p* < 0.05 was significant. *refer to significant levels at <5%. NPA, nasopharyngeal aspirate; NW, normal weight; OW, overweight; O, obese.

No significant differences were seen between leptin level and nutritional status in both plasma and NPA samples (Figure 3). On the other hand, we analyzed NPA and plasma leptin level in children according to nutritional status separating by age group analyzed above (less or greater than 6 months) (Figure 4). We found a higher plasma leptin level in obese children <6 months than normal weight children <6 months (Figure 4C; 7.58 vs. 5.12 ng/μl; *p* < 0.046).

## Discussion

Obesity and ALRIs constitute two important morbidity factors in the world, and its relationship began to be studied after the influenza H1N1 pandemic (7, 8). In a multivariate analysis, we found that obese and overweight infants <6 months old were associated with disease severity as defined by longer hospitalization and oxygen therapy treatment. Some previous studies have examined the impact of nutritional status on respiratory infections in children. Similar to our results, Rivera-Claros et al. concluded that obesity is a risk factor for worse clinical course of acute lower respiratory tract infections in Chilean infants with RSV infections and without chronic disease (9). A study of 1,129 Polish children found that overweight children had a higher risk for acute upper respiratory tract infections (22). Another observational study of 1,116 children hospitalized with community-acquired pneumonia in the United States, found that overweight or obese children were more likely to be admitted to the ICU (23). However, it is still unclear why children with overnutrition have proven to be more prone to have severe respiratory infections.

We found that in the group of children <6 months, plasma leptin level of obese patients was higher than normal weight patients. It is described that the adipose tissue acts as an endocrine organ, producing adipokines that exert immunomodulatory effects. Leptin is a pro-inflammatory adipokine produced primarily in adipose tissue that increases in proportion to the body adiposity. The primary function of leptin is the control of appetite via the hypothalamus, and it has been established as a key hormone in deregulation of immune responses in obese patients (24). Teran-Cabanillas et al. (13) showed that leptin induces SOCS3 overexpression which leads to reduced type I IFN production in obese patients. Downstream in the route of type I IFNs, the antiviral 2′-5′-oligoadenylate synthetase 1 (OAS1) gene is also activated via JAK-STAT. We analyzed a small number of samples available for expression of *OAS1* gene. Despite not finding significant differences, we found a tendency to decrease the expression of the *OAS1* gene in overweight and obese patients compared to normal weight children (Data not shown). Some studies have related leptin level with hepatitis virus infection. In the study by Caner et al. (25) leptin levels were found unaltered in children with acute hepatitis A. However, Tóth et al. (26) showed an association between the changes of serum leptin levels in children and the severity of hepatitis disease. It remains to be determined whether the infection by respiratory viruses affects leptin levels.

We analyzed plasma and NPA leptin level according to age (data not shown). We found that children <6 months showed higher plasma leptin level than older children, regardless of nutritional status (5.26 vs. 1.29 ng/μl; *p* < 0.0001). Most of children <6 months (69%) were exclusive breastfeeding at the time of sampling. Savino et al. (27) observed a positive association of breast milk leptin values not only with maternal body mass index but also with maternal and infant serum leptin values. On the other hand, it has been described that infants have an increased risk of obesity later in life if they have an overweight or obese mother (28) which can be reduced with the use of low-protein feedings after 3 months (29). Chile has one of the highest prevalence rates of overweight and obesity in Latin America (30). Overweight has 39% prevalence and obesity has 29%; 51% of women in reproductive age have a state of overnutrition (31). According to this, we speculate that the excess weight of mothers causes an increase of leptin in children under 6 months who receive breastfeeding, and this could have an impact on the severity of viral infections. Therefore, further studies are needed to study the effect of breast milk leptin in infants.

Here, we have shown for the first time to our knowledge, that obesity and overweight conditions are associated with viral coinfections in children hospitalized for LRTIs. Although it is still unclear why this is so, there is some evidence that can start to shed light on this association. It has been reported that the erythrocyte membrane responds very early to modifications of plasma lipoproteins and suggest that in childhood obesity a modified transfer of cholesterol from plasma to erythrocyte membrane may take place (16). Several viruses use cholesterol-rich microdomains (lipid rafts) to infect host cells (32–34), as has been shown in influenza virus infections. Influenza A virus rafts serve as a site for concentrating viral proteins and promoting the re-production of infectious viruses (35). Moreover, it has been reported that RSV uses lipid rafts in the plasma membrane as attachment platforms to enter normal human bronchial epithelial cells (36). According to this, it is possible that the membrane composition of the respiratory tract cells of children with overnutrition is modified in a way that favors the entry of respiratory viruses. It has long been known that coinfections exhibit a phenomenon called viral interference, where one virus blocks the growth of another (37), thus the high frequency of simultaneous respiratory infections in obese and overweight patients is somewhat surprising and needs further study. Consequently, we believe that overnutrition in children could alter the composition of cholesterol in the membrane of the respiratory epithelial tissue, facilitating the entry of viruses in different cells, which could explain the results obtained in this study. An inadequate immune response might also allow a greater entry of these pathogens. Obesity provokes an imbalance in the immune system, including an aberrant type I interferon response, which are the key cytokines involved in the early immune response to viral infections (38). The increase in coinfection related to obesity and overweight conditions may have additional health effects. It was recently reported that asthma in children between 6 and 8 years old is more frequent and severe in those that have been previously hospitalized with viral coinfection-bronchiolitis, compared to those with single infections (39). The long-term effects of metabolic changes caused by early onset obesity must be explored in follow-up studies.

Obese and overweight patients, in addition to exhibiting high levels of viral coinfections as well as more severe infections, also presented lower viral loads than infants with standard weights. Through a mathematical model, HRV, the fastest-growing virus, reduces the replication of the remaining viruses during a coinfection, while PIV, the slowest-growing virus is suppressed in the presence of other viruses (40). According to this, coinfections could limit the increase of RSV load because of competition for resources with other viruses. These results are in agreement with those previously described by Garcia-Mauriño (41), where high viral load was associated with less severe RSV in children.

A possible limitation of our study is that we analyzed NPA samples using the RT-PCR method. It is unknown if this highly sensitive molecular technology detects only pathogens that cause LRTIs or if some of the viruses detected could be commensal pathogens (42). Some of these detections might represent post infectious shedding. We addressed and controlled this problem by excluding children that had suffered from respiratory infections 2 weeks prior to the study.

Effective interventions are required to reduce childhood obesity. Meanwhile, further research is needed to determine the role of leptin and other immunological markers in the severity of viral infection and to understand the pathophysiology of viral coinfection as it relates to nutritional status as well as to explore the epithelial-specific response to respiratory viruses in primary cells of lean and obese child.

## Data Availability Statement

The datasets generated for this study are available on request to the corresponding author.

## Ethics Statement

The studies involving human participants were reviewed and approved by Scientific Ethics Committee, Universidad Autónoma de Chile. Written informed consent to participate in this study was provided by the participants' legal guardian/next of kin.

## Author Contributions

The concept and design of the study was carried out by LF, GA-B, JI, DG-D, and GV. LF, GA-B, and GV obtained the clinical data. GA-B and MR-F conducted the experimental analysis. LF and FZ-R carried out the statistical analysis. LF, GA-B, and FZ-R wrote the manuscript. All of the authors contributed to the interpretation of the data and critically revised the manuscript, providing important intellectual content and approving the final report.

### Conflict of Interest

The authors declare that the research was conducted in the absence of any commercial or financial relationships that could be construed as a potential conflict of interest.
