# Supplementary figures and images for: Overnutrition in Infants Is Associated With High Level of Leptin, Viral Coinfection and Increased Severity of Respiratory Infections: A Cross-Sectional Study
Source: Front Pediatr. 2020 Feb 18;8:44. doi: 10.3389/fped.2020.00044 (PMC7041426; doi:10.3389/fped.2020.00044)

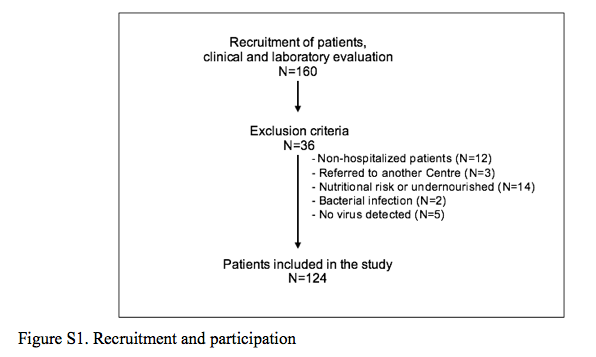

Supplement: Supplementary file 1 [file Image_1.TIFF]

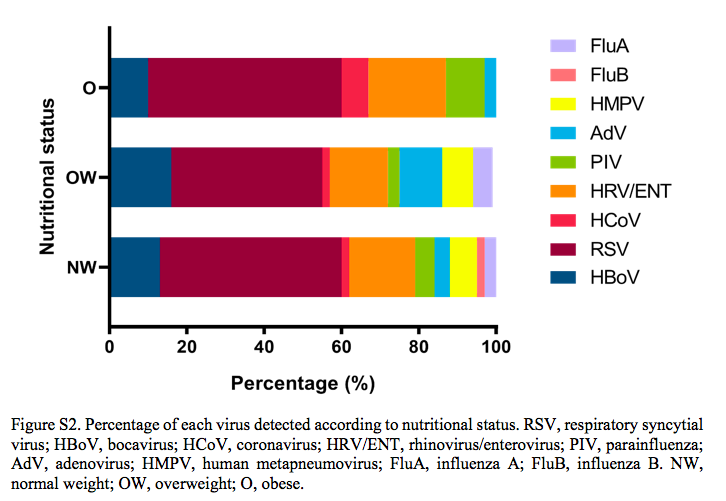

Supplement: Supplementary file 2 [file Image_2.TIFF]
